# First evidence of conspecific hyperparasitism in *Dermacentor marginatus* nymphs feeding on a rabbit under experimental conditions

**DOI:** 10.1007/s10493-025-01103-w

**Published:** 2026-01-15

**Authors:** Lenka Minichová, Ľubomír Vidlička, Mirko Slovák

**Affiliations:** 1 Institute of Microbiology, Czech Academy of Sciences, Prague, Czech Republic; 2 Institute of Virology, Biomedical Research Center, Slovak Academy of Sciences, Bratislava, Slovakia; 3 Institute of Zoology, Slovak Academy of Sciences, Bratislava, Slovakia

**Keywords:** Hyperparasitism, *Dermacentor marginatus*, Nymphs, Ixodidae, Tick colony

## Abstract

Hyperparasitism in ticks, particularly in nymphs of the order Ixodidae, is a rare phenomenon. In our laboratory tick colony, female rabbits are used as a blood source for the ticks, housing them under controlled conditions. Feeding *Dermacentor marginatus* nymphs monitoring was performed daily, and engorged and detached ticks were collected and stored in desiccators at constantly 24 ± 2 °C and 85–90% relative humidity (16 h light/8 hours dark). Nymphs suspected of conspecific hyperparasitism were preserved in ethanol for analysis and imaging. This study presents the first documented case of hyperparasitism in *D. marginatus* and contributes to the limited literature on hyperparasitism in Ixodidae nymphs. While such cases are observed in controlled tick colonies, their occurrence in the wild, especially in Ixodidae, is extremely rare compared to Argasidae ticks. Furthermore, the frequency of this phenomenon in the wild and its possible eco-epidemiological significance remain poorly understood.

## Introduction

*Dermacentor marginatus* (Sulzer 1776), also known as ornate sheep tick, infests domestic (sheep, dogs, goats, horses, cattle) and wild hosts including deer, hare, hedgehog, wild boar, and wolf (Accorsi et al. 2022; Estrada-Peña et al. 2017; Garcia-Vozmediano et al. 2020; Rubel et al. 2016; Sgroi et al. 2021). It is adapted to a warmer and drier climate within the belt of 33–51° N latitude and generally inhabits lowlands, steppes (alpine and forest), and semi-desert areas (Rubel et al. 2016; Walter et al. 2016). This three-host tick species has a life cycle of 75–163 days under laboratory conditions, but in nature it usually completes its cycle within 1–2 years (Kahl and Dautel 2013; Nosek 1972). Larvae are commonly found on rodents and small to medium-sized insectivores (Estrada-Peña et al. 2017; Nosek 1972). Adult female ticks have been found on humans, including children, particularly on the scalp (Cazorla et al. 2003; Porta et al. 2008; Raoult et al. 2002; Selmi et al. 2008).

*Dermacentor marginatus* is associated with the occurrence of numerous tick-borne pathogens (Bonnet et al. 2013; Estrada-Peña et al. 2017; Sonenshine and Roe 2014), some of which are recognised as increasingly important veterinary and public threat (e.g. tick- borne encephalitis virus, *Rickettsia slovaca*, *Rickettsia raoultii*) (Buczek et al. 2020; Garcia-Vozmediano et al. 2020; Ličková et al. 2025; Nadim et al. 2021; Nosek and Kozuch 1985; Rubel et al. 2016; Špitalská et al. 2012).

Hyperparasitism describes a phenomenon where a tick parasitises another tick feeding on a host. It involves non-fed ticks attaching to fed or feeding ticks of the same (intraspecific, conspecific) (Buczek et al. 2019) or different species/genus (interspecific, heterogeneric) (Uspensky 2024). This behaviour was previously misclassified as cannibalism or kleptoparasitism, but Uspensky later clarified the correct terminology (Uspensky 2023).

The first documented case of hyperparasitism (unfed male of *Amblyomma variegatum* attached conspecific to a feeding female) was recorded by Barber in 1895 (Barber 1895). Buczek compiled a comprehensive review of hyperparasitism in both soft and hard ticks, including a detailed table on host species, engorgement level, and tick distribution (Buczek et al. 2019). In the most recent review, Uspensky has developed this topic further and provides an in-depth analysis of both the biological and eco-epidemiological aspects of hyperparasitism (Uspensky 2024). Here we report the first case of hyperparasitism in nymphs of *D. marginatus* under laboratory conditions and provide a detailed description of this phenomenon.

## Materials and methods

In our artificial laboratory breeding of tick colonies, all tick stages are usually fed on female California rabbits obtained from the Research Institute for Animal Production (Nitra, Slovakia). Animals were housed individually in cages in the animal facility of the Institute of Virology, Biomedical Research Center of the Slovak Academy of Sciences under controlled conditions: 15–21 °C, 45–65% relative humidity, and photoperiod 12 h light/12 hours dark cycle. All animals were fed standard pellet diet and given water ad libitum. All procedures followed protocols approved by the Animal Use Protocol approved by the ethical committee of Biomedical Research Center of Slovak Academy of Sciences and the State Veterinary and Food Administration of the Slovak Republic (facility number SK UCH01016, permit number 3954/19–221 and 292/16–221 k).

The rabbit’s fur was partially shaved on the back to attach neoprene chambers containing ticks. For more details, please see Slovák et al. (2002). Ticks of each developmental stage were placed separately in individual chambers and on separate animals. Depending on the life stage of ticks, we checked when fully engorged ticks are ready to detach: Larvae were checked twice a day, while nymphs and adult ticks were checked once a day. The collected fully engorged ticks were stored in desiccators at 24 ± 2 °C, 85–90% relative humidity, and a photoperiod of 16 h light and 8 h dark cycle.

Nymphs suspected of being hyperparasitic were placed in 70% ethanol and examined under a Leica M205 C stereomicroscope equipped with a Leica flexacam C3 camera, and the images were processed using Adobe Photoshop CS6.

## Results

**Fig. 1:**
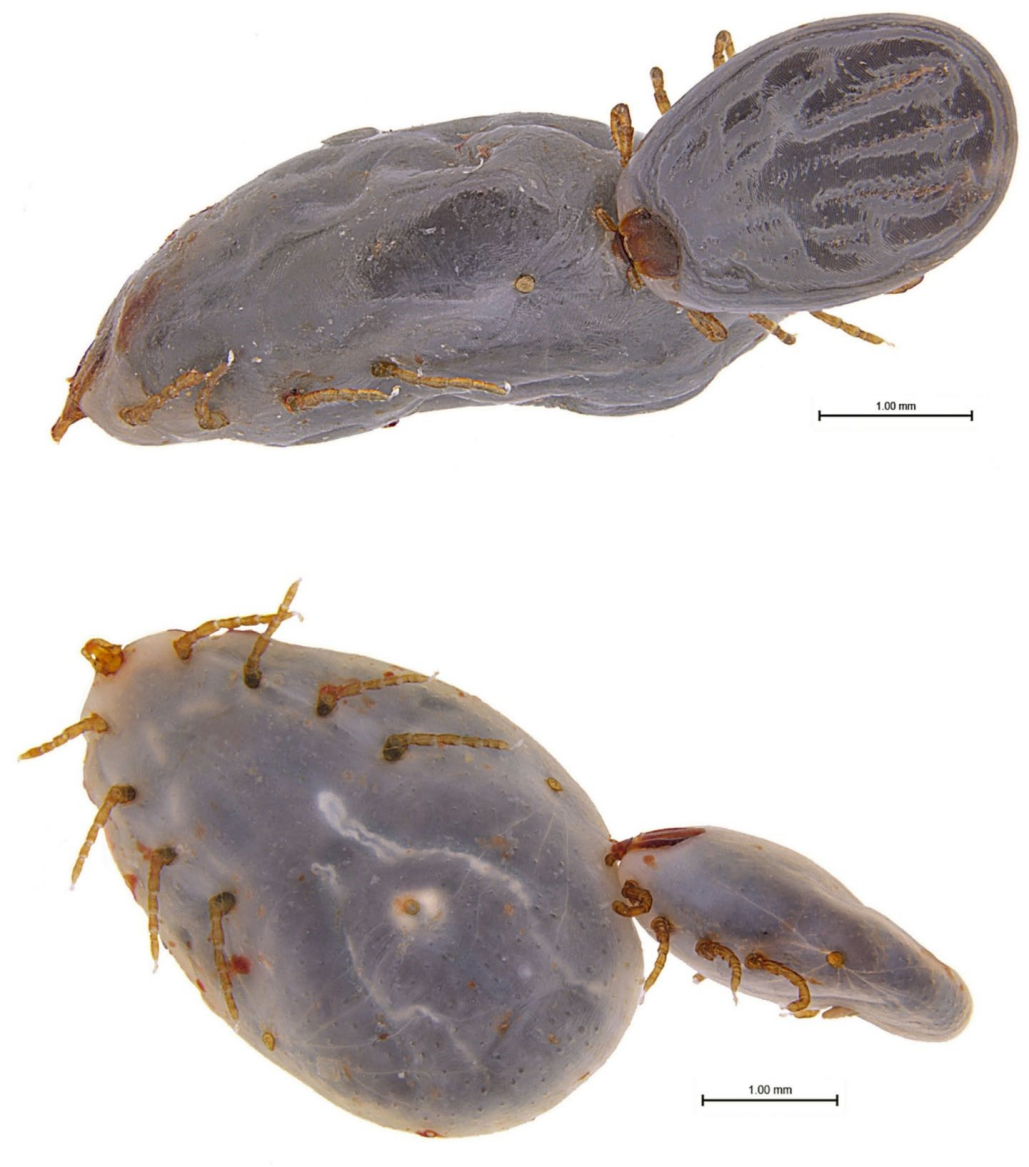
Hyperparasitism of *D. marginatus* nymphs (parasitized nymph on the left, hyperparasitic nymph on the right)

The nymphs of *D. marginatus* (about 500 individuals per chamber) were allowed to feed on a rabbit, and fully engorged nymphs were collected daily. On the sixth day of feeding, we observed two nymphs, one of which was attached to the other. A close examination under the stereomicroscope showed the perfect anchoring of the hypostome of one nymph in the integument of the other, laterally and caudally, near the spiracle (Fig. 1). It can be clearly seen that the nymph has opened its palps, and its hypostome is embedded in the body of the other nymph, which is a case of conspecific hyperparasitism. To our knowledge, this is the first report of conspecific hyperparasitism in *D. marginatus*. It is also one of the few cases of hyperparasitism in tick nymphs.

## Discussion

As summarized by Buczek et al. (2019) and Uspensky (2024), cases of hyperparasitism are mainly known from tick colonies under laboratory conditions. In argasid ticks, hyperparasitism has been observed primarily during laboratory rearing or in field-collected specimens brought into laboratory conditions. It typically involves unfed individuals stealing blood from engorged individuals, often as a response to starvation or overcrowding. Uspensky reported the occurrence of this phenomenon in nature is extremely rare, and although tick-to-tick feeding among argasid ticks has never been observed under field conditions, there is evidence of this behaviour in the form of scars on the cuticle of free-living ticks (Uspensky 2024). In *Ixodes* ticks, hyperparasitism has been documented both in laboratory settings and among field-collected individuals. In this genus, male hyperparasitism on females is likely associated with mating attempts, whereas in Amblyomminae, it may represent an aberrant feeding behaviour (Buczek et al. 2019; Uspensky 2024). The author even mentions occasional observations of ixodid ticks attaching to horseflies and cites three relevant sources in Russian (Uspensky 2024).

The comprehensive reviews by Buczek et al. (2019) and Rodrigues et al. (2023) show that hyperparasitism in ticks from the families Ixodidae and Argasidae occurs predominantly in the form of males parasitizing females (Buczek et al. 2019; Rodrigues et al. 2023). Cases of nymphal hyperparasitism have been summarized in review of Buczek et al. in the Argasidae, particularly in *Ornithodoros turicata*,* Ornithodoros parkeri* and *Ornithodoros erraticus*, and in the Ixodidae, especially in *Hyalomma detritum* (Buczek et al. 2019). According to Uspensky, the hyperparasitism of males on unfed or fed females in case of *Ixodes* ticks is a side effect of mating, whereas in Metastriata ticks, it appears to be a rare feeding aberration (Uspensky 2024).

One potential factor contributing to nymphal hyperparasitism could be overcrowding, i.e. a high number of nymphs per chamber. However, in our observations, a different pattern emerged: despite ample space within the chamber, nymphs consistently clustered in one half, leaving the other half unoccupied. A similar aggregtion behaviour was described by Wang et al. in *Rhipicephalus appendiculatus*, where ticks gathered in a single area despite the availability of space. This behaviour was linked to the presence of immunoglobulin-binding proteins (IGBPs), particularly the male-specific IGBP-MC, which is secreted in saliva and facilitates female feeding. Males remain near females after mating, likely to enhance their feeding success and thereby increase their own reproductive fitness—a behaviour described as mate guarding (Wang and Nuttall 1998).

Hyperparasitism may enable ticks to ingest haemolymph or vertebrate blood from conspecific or heterospecific hosts, potentially influencing intra-tick pathogen transmission. Williamson and Schwan (2018) demonstrated that unfed males of *Ornithodoros hermsi* frequently parasitize engorged nymphs and are capable of acquiring and transmitting *Borrelia hermsii*, indicating a possible role of hyperparasitism in the enzootic maintenance of this pathogen (Williamson and Schwan 2018). These findings highlight the need for further investigation into the ecological relevance of hyperparasitism in natural tick populations.

We support the hypothesis proposed by Uspensky that the transmission of pathogens by hyperparasitism is theoretically possible also in hard ticks. Labruna et al. consider this a possible alternative mechanism for the transmission of microorganisms among ticks (Labruna et al. 2007; Uspensky 2024). However, as Uspensky further emphasized, there is no evidence for its occurrence (Uspensky 2024). Therefore, the potential eco-epidemiological relevance of this phenomenon in hard ticks is still uncertain.

## Conclusion

This case illustrates a rare occurrence of conspecific hyperparasitism in hard ticks that is usually reported in controlled laboratory settings. The ecological and evolutionary implications of this behaviour and its possible role in the transmission dynamics of tick-borne diseases are not yet fully understood. Further studies are needed to investigate how such behaviour might influence the transmission of pathogens in hyperparasitism relationships and to expand our knowledge of the eco-epidemiology of tick-borne diseases.

## Data Availability

We declare all data is being provided within this manuscript.
